# Pyruvate Kinase M2 Expression, but Not Pyruvate Kinase Activity, Is Up-Regulated in a Grade-Specific Manner in Human Glioma

**DOI:** 10.1371/journal.pone.0057610

**Published:** 2013-02-25

**Authors:** Joydeep Mukherjee, Joanna J. Phillips, Shichun Zheng, John Wiencke, Sabrina M. Ronen, Russell O. Pieper

**Affiliations:** 1 Department of Neurological Surgery, University of California San Francisco, San Francisco, California, United States of America; 2 Department of Pathology, University of California San Francisco, San Francisco, California, United States of America; 3 Department of Radiology and Biomedical Imaging, University of California San Francisco, San Francisco, California, United States of America; 4 The Brain Tumor Research Center, University of California San Francisco, San Francisco, California, United States of America; Wake Forest University, United States of America

## Abstract

Normal tissues express the M1 isoform of pyruvate kinase (PK) that helps generate and funnel pyruvate into the mitochondria for ATP production. Tumors, in contrast, express the less active PKM2 isoform, which limits pyruvate production and spares glycolytic intermediates for the generation of macromolecules needed for proliferation. Although high PKM2 expression and low PK activity are considered defining features of tumors, very little is known about how PKM expression and PK activity change along the continuum from low grade to high grade tumors, and how these changes relate to tumor growth. To address this issue, we measured PKM isoform expression and PK activity in normal brain, neural progenitor cells, and in a series of over 100 astrocytomas ranging from benign grade I pilocytic astrocytomas to highly aggressive grade IV glioblastoma multiforme (GBM). All glioma exhibited comparably reduced levels of PKM1 expression and PK activity relative to normal brain. In contrast, while grade I-III gliomas all had modestly increased levels of PKM2 RNA and protein expression relative to normal brain, GBM, regardless of whether they arose *de novo* or progressed from lower grade tumors, showed a 3–5 fold further increase in PKM2 RNA and protein expression. Low levels of PKM1 expression and PK activity were important for cell growth as PKM1 over-expression and the accompanying increases in PK activity slowed the growth of GBM cells. The increased expression of PKM2, however, was also important, because shRNA-mediated PKM2 knockdown decreased total PKM2 and the already low levels of PK activity, but paradoxically also limited cell growth *in vitro* and *in vivo*. These results show that pyruvate kinase M expression, but not pyruvate kinase activity, is regulated in a grade-specific manner in glioma, but that changes in both PK activity and PKM2 expression contribute to growth of GBM.

## Introduction

Tumor cell metabolism differs from normal cell metabolism in ways that have broad consequences for our understanding of the tumorigenic process. Normal non-proliferative cells under oxygenated conditions convert glucose to pyruvate, then move pyruvate into the mitochondria where processing in the citric acid cycle generates the reducing equivalents necessary for oxidative phosphorylation and ATP generation [1], [2]. Under hypoxic conditions, normal cells shunt pyruvate away from the mitochondria and converted it to lactate. This anaerobic glycolysis supports energy production but at a much lower level than under oxygenated conditions [3]. Proliferating tumor cells most resemble hypoxic cells in that they favor conversion of pyruvate to lactate. Tumor cells, however, convert pyruvate to lactate even under oxygenated conditions in a process referred to as aerobic glycolysis [4], [5]. This so-called “Warburg effect” limits energy production, but is also widely thought to provide conditions that favor tumor growth, and Warburg himself suggested that the metabolic shift noted in cancer was a driving force in the disease [4].

A major advance in our understanding of the factors that regulate tumor cell metabolism was the discovery that the tumor-associated shift to aerobic glycolysis was controlled by the balance of expression of 2 isoforms of the glycolytic enzyme pyruvate kinase M (PKM)[6]. Pyruvate kinase converts phosphoenolpyruvate (PEP) to pyruvate and is a rate-limiting enzyme in glycolysis [7]. PKM is one of three PKs (PKM, PKL, which is expressed only in the liver, and PKR which is expressed only in erythrocytes)[8] but the only form associated with cancer. The two isoforms of PKM are encoded by a single gene on chromosome 15, and are derived from a single transcript that gives rise to PKM1 mRNA and PKM2 mRNA by differential splicing [9]. The processed PKM1 mRNA excludes exon 10 while the processed PKM2 mRNA excludes exon 9, making the two transcripts best distinguishable by quantitative PCR. The PKM1 protein is constitutively active, has a high affinity for its substrate PEP, converts PEP to pyruvate, and through complexation with other enzymes of the glycolytic pathway assures that pyruvate is moved into the mitochondria for use in the citric acid cycle [10]. Although PKM1 is frequently stated to be expressed in most adult normal tissues, the most stringent analyses suggest that it is expressed only in tissues in which a large amount of energy is produced such as muscle and brain [11]. In these tissues PKM1 expression is associated with a high PK activity and the oxidative phosphorylation that helps generate maximal amounts of ATP. The PKM2 protein in contrast is expressed in some differentiated tissues such as lung, but primarily is expressed in embryonic tissues and tumor cells [8], [12], [13]. PKM2 can exist in active tetrameric and nearly inactive dimeric forms although in tumors it is reported to exist nearly exclusively as a dimer [14]. The dimeric form of PKM2 has a low affinity for PEP, is not associated with enzymes of the glycolytic complex, and favors conversion of pyruvate to lactate [7], [15]. As a result of the inability of the PKM2 dimer to stimulate pyruvate production, glycolysis slows and the intermediates of glucose metabolism back-up in tumor cells. These intermediates in turn are used to generate the nucleotides and amino acids that are required for cell proliferation, increased biomass, and tumor growth [16], [17]. Tumor formation therefore appears to involve alterations in PKM isoform expression, accompanied by a tumor-promoting shift in PK activity and metabolism from oxidative phosphorylation to aerobic glycolysis [6], [18].

Although tumors in general are thought to exhibit high levels of PKM2 and low levels of PK activity, this belief is based primarily on PKM2 RNA, PKM2 protein, or PK activity analysis (but rarely all three) of a limited number of high grade tumors. As a result, there is little information as to how changes in PKM mRNA levels relate to changes in PKM protein levels and PK activity, whether PKM isoform expression and activity changes along a continuum of grades tumors of the same histologic subtype, and what the consequences of these changes might be for tumor growth. To address these questions we examined PKM isoform expression and PK activity in an extensive series of grade I-IV astrocytomas, and have examined the consequences of alteration of PKM expression and activity on the growth of human glioma cells. The results of the studies show that while PK activity is uniformly low in all glioma, PKM2 expression is disproportionately up-regulated in GBM, and that both these events play roles in glioma growth.

## Materials and Methods

### Ethics Statement

All animal work was conducted according to the guidelines set forth by the UCSF Institutional Animal Care and Use Committee(IACUC) in accordance with the recommendations of the Weatherall report, “The use of non-human primates in research”, and was approved by the UCSF IACUC.

### Normal brain and brain tumor samples

All specimens were obtained from the UCSF Brain Tumor Research Center Tissue Core (University of California, San Francisco) using protocols approved by the UCSF Institutional Review Board. Non-tumor brain tissue samples were obtained from autopsy (N = 2) or from cancer-free epilepsy patients who underwent temporal lobe resection (N = 11). Uncultured human NG2(+)/A2B5(+) human neural progenitor cells were obtained from Arturo Alvarez-Buylla (University of California-San Francisco). Formalin-fixed paraffin embedded sections were used for neuropathological verification of tumor grade based on the WHO classification scheme [19] and identified grade I (juvenile pilocytic astrocytoma, N = 10, adult pilocytic astrocytoma, N = 14), grade II (diffuse astrocytoma, N = 15), grade III (anaplastic astrocytoma, N = 25), and grade IV (glioblastoma, N = 38) specimens. DNA, mRNA and protein were isolated from corresponding frozen and fixed sections that contained greater than 75% non-necrotic tumor tissue. All WHO grade IV specimens were analyzed for IDH1/2 mutations as previously described [20] and those with a previous histological diagnosis of a lower-grade glioma in combination with IDH mutation were defined as secondary GBM (N = 17).

### Cell Culture

U87 and T98 GBM cells (American Type Culture Collection) were maintained in DMEM-H21 supplemented with 10% fetal bovine serum (JRScientific) at 37 °C in a humidified 5% CO2 incubator.

### Quantitative PCR analysis

Total RNA was isolated from samples (Qiagen RNeasy Kit), treated with DNase I (0.4 units/µg RNA), reverse transcribed (SuperScript II Reverse Transcriptase Kit, Invitrogen) and amplified (initial denaturation of 95 °C for 3 min followed by 40 cycles at 95 °C for 15sec, 60 °C for 15 sec, and 72 °C for 30 sec, Chromo4 Real Time PCR Detector, MJ Research) in triplicate using primers designed to bind to PKM1-specific exon 9 (f-ACCGCAAGCTGTTTGAAGAA and r-TCCATGAGGTCTGTGGAGTG) or PKM2-specific exon 10 (f-GAGGCCTCCTTCAAGTGCT and r-CCAGACTTGGTGAGGACGAT), sequences, respectively. The mean Ct values for each set of amplifications were determined, after which the mean Ct value from triplicate qPCR reactions using primers specific for the housekeeping gene HPRT1 (IDT) were subtracted to derive a ΔCt value. Relative mRNA expression was expressed as 2 ^ΔCt^. The negligible variance of HPRT1 Ct values across all samples analyzed (17.4 ± 0.4) allowed direct comparison of PKM1 and PKM2 mRNA isoform expression within and between samples.

### PKM Splicing assay

A PKM splicing assay was adopted from [21]. RNA isolated from normal or tumor samples (fixed or frozen) was reverse transcribed then amplified (95 °C for 2 min followed by 94 °C for 30sec, 55 °C for 1 min, 72 °C for 90 sec for 40 cycles) using a forward primer that bound to shared PKM1/2 exon 8 and a reverse primer that bound to shared PKM1/2 exon 11 sequences [20]. The 442 bp PCR products representing both PKM1 and PKM2 transcripts were then digested with PstI and electrophoresed, after which the 442 bp (uncut PKM1-specific amplicon) and 246 bp and 196 bp (PstI-cleaved PKM2-specific amplicon) products were quantitated (Image-J software). Sham-digested PCR products were used as a control for the PCR amplification while PstI-digested amplification products derived using a PKM2 cDNA template were used as a control for restriction enzyme digestion.

### Immunohistochemistry

Immunohistochemistry was performed on a Ventana Medical Systems Benchmark XT using anti-human PKM1 (1:800 dilution, 60 min, ProteinTech) or PKM2 (1:100 dilution, 32 min, Schebo Bio) antibodies. Staining was visualized using 3, 3′-Diaminobenzidine tetrahydrochloride (Ventana). Negative and positive controls, confirmed by Western blotting, were included in each run. Staining was scored using a four-tier scale: 0, no immunostaining; 1, >0 and < 10% positive; 2, between 10 and 25% positive; 3, >25 and < or  = 75%; and 3, >75% tumor cells positive.

### Modulation of PKM isoform expression

#### PKM1

U87 or T98 cells (1×10^6^) were transfected with PKM1-pCDNA3.1 [22] or pCDNA 3.1 (negative control) using Fugene-6 transfection reagent (Roche). After 2 weeks of G418 selection (1 mg/ml, Roche), 6 colonies were picked, expanded in G418-containing medium, and assessed for PKM1 expression by Western blot analysis. Two PKM1-expressing clonal populations were chosen for further study.

#### PKM2

Scramble or one of five different PKM2 shRNA constructs (pLKO.1, Open Biosystems) were co-transfected along with VSV-G and ΔVPR plasmids (Open Biosystems) at a 1:0.9:0.1 ratio into 293T packaging cells using Fugene-6 (Roche). Lentiviral supernatants were harvested at 24 and 48 hours post-transfection and used to infect U87 and T98 cells for 24 hours in the presence of polybrene (8 µg/ml, Sigma). Following puromycin selection (1 µg/ml, two weeks) and expansion, the 2 clonal populations exhibiting the lowest PKM2 expression relative to scramble controls were chosen for further study.

#### Proliferation, cell cycle distribution, and clonogenic assays

Proliferation was assessed using Alamar Blue reagent (Invitrogen). For cell cycle distribution, fixed, propidium iodide-labeled cells were subjected to flow cytometry using a FACSCalibur (BD Biosciences) in combination with Flowjo software (Treestar)[23]. Clonogenic assays were performed as previously described [24]. Numbers of colonies (>50 cells) and average diameter of the colonies for each condition were measured on 100× photomicrographs and analyzed using Metamorph Imaging Software (Molecular Devices).

#### Biochemical assays

Pyruvate kinase activity and intracellular concentrations of pyruvate and lactate were measured using pyruvate kinase, pyruvate or lactate assay kits (BioVision). Intracellular levels of ATP were measured using the ATPlite 1Step Luminescence Assay System® (PerkinElmer) as per the manufacturer's protocol. Six reactions were performed per sample.

#### Protein extraction and Western blot analysis

Protein lysates from GBM cell lines and frozen operative specimens were prepared in lysis buffer (50 mM HEPES, pH7.0, 150 mM NaCl, 10% Glycerol, 1% Triton-X, 1 mM EDTA, 100 mM NAF, 10 mM NaPPi) supplemented with protease and phosphatase inhibitors (Roche). Proteins were separated on 4–20% Tris-Glycine gradient polyacrylamide gels (Invitrogen) and transferred onto Immuno-Blot PVDF membranes (Bio-Rad Laboratories). Membranes were then incubated in blocking buffer (1X TBS containing 5% milk and 0.05% Tween-20, 2 hours), probed overnight with antibodies specific for PKM1 (Proteintech, 1:1000), PKM2 (Cell Signaling, 1:1000) or β-actin (Cell Signaling, 1:20,000), washed, then incubated with appropriate horseradish peroxidase-conjugated secondary antibodies (Santa Cruz Biotechnology). Antibody binding was detected by incubation with ECL reagents (Amersham Pharmacia Biotech).

#### Intracranial tumor formation

Immunodeficient mice (nu/nu; Charles River) were injected intracranially with 4×10^5^ luciferase-expressing U87-Scr-Luc (N = 5) or U87-shPK-M2-Luc (N = 5) cells as described [25]. Tumor growth was monitored weekly by treating mice with D-luciferin (150 mg/kg IP, Gold-Biotechnology) and measuring bioluminescence using a Xenogen IVIS Bioluminescence imaging station (Caliper). Tumor growth was calculated by normalizing luminescence measurements to day-1 post injection values. Animals were monitored daily until they developed signs of neurological deficit, at which time they were sacrificed.

#### Statistical analysis

When two groups were compared, the unpaired Student's t test was applied (P-value). When multiple groups were evaluated, the one-way ANOVA test with post hoc Turkey-Kramer multiple comparisons test was used.

## Results

### PKM expression, but not PK activity, is modulated in a grade-specific manner in human glioma

**Figure 1:**
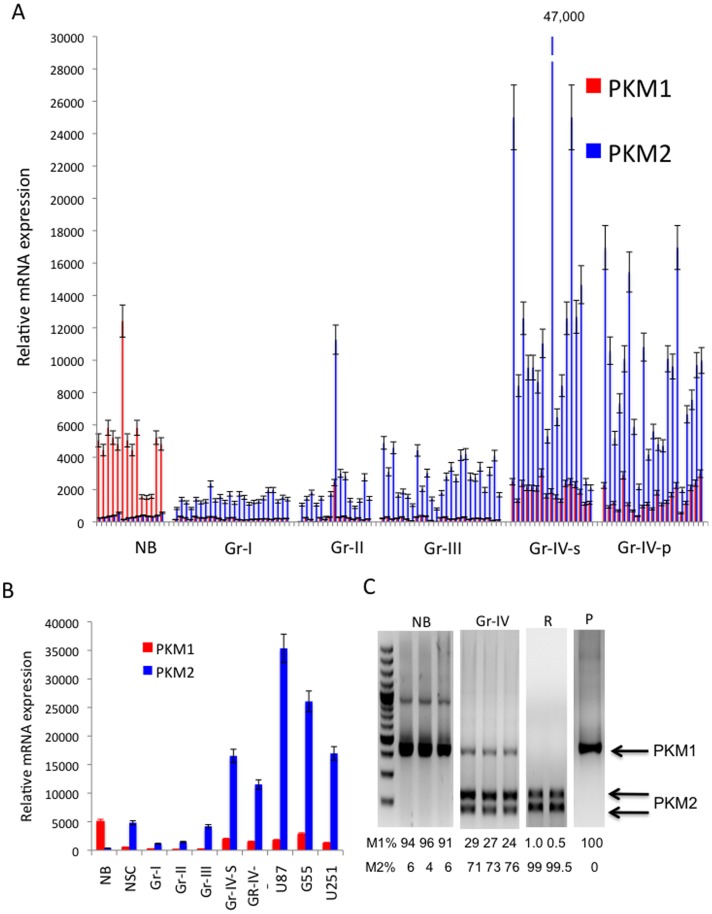
PKM1 and PKM2 mRNA expression in a series of human normal brain (NB), neural progenitor cells (NSC), and WHO grade I-IV human astrocytoma specimens. A, RNA was isolated from fixed or frozen normal brain (NB) and grade (Gr)- I, II, III, and IV (primary, P and secondary, S) astrocytoma samples, reverse transcribed, then subjected to triplicate qPCR analysis using primers specific for the PKM1 or PKM2 transcript. All values are the mean normalized to HPRT1 expression. B, mean group PKM1 and PKM2 mRNA expression values from panel A and from NSC and established GBM cell lines. C, cDNAs from representative samples in panel A were subjected to PCR amplification using primers amplifying a 442 bp exon 8–11 region common to PKM1 and PKM2. Following incubation with PstI, the uncleaved (PKM1, 442 bp) and cleaved (PKM2, 246 and 196 bp) amplification products were separated by electrophoresis and quantitated, with total signal (PKM1+PKM2) set at 100 for each lane. P, PCR control (Gr-IV amplification products prior to PstI digestion); R, duplicate restriction enzyme controls (amplification products derived using a PKM2 cDNA template post-PstI digestion).

To begin to examine the uniformity and relevance of PKM isoform expression and activity, we first wished to analyze PKM mRNA isoform expression in formalin-fixed or frozen normal human brain and WHO grade I–IV astrocytomas. This information is not readily available in public databases which typically contain data generated using methodology that fails to distinguish PKM1 from PKM2 expression. We therefore used quantitative PCR analysis using primers specific for PKM exon 9 (PKM1) or PKM exon 10 (PKM2) in combination with control primers that amplified HPRT mRNA and allowed for normalization of the results. As shown in Fig 1A and B, all normal brain samples expressed high levels of PKM1 transcript, while levels of PKM2 mRNA expression were detectable, but nearly 10-fold less than that those of PKM1 mRNA. These results were consistent with previous data suggesting that PKM1 is the predominant PKM isoform expressed in normal brain and served as a positive control for studies in glioma [11]. In contrast to normal brain, WHO grade I astrocytomas, which are by definition benign lesions, expressed roughly 90% less PKM1 mRNA. These tumors also exhibited a small but statistically significant increase in PKM2 expression relative to normal brain. Both grade II and grade III tumors were statistically indistinguishable from grade I tumors with regard to PKM1 and PKM2 mRNA expression, despite the fact that grade III tumors are considered to be high-grade lesions. Furthermore, the PKM1 and PKM 2 expression of grade I, II, or III lesions were not statistically different from that of a human neural progenitor cell population. In contrast, GBM (WHO grade IV astrocytoma) differed from other gliomas in that they expressed levels of PKM2 mRNA 3–5 times that noted even in the grade III gliomas. The same changes were noted regardless of whether the GBM samples assessed were derived from primary, *de novo* GBM (as defined by lack of IDH mutation, Gr-IV-p), IDH mutant secondary GBM that arose from lower grade gliomas (Gr-IV-s)[26], or GBM cells in culture.

To verify the results derived from qPCR, the relative expression of PKM1 to PKM2 transcripts in each given sample was also assessed by a PCR-based assay using primers that amplified all PKM1 and PKM2 transcripts, followed by a restriction enzyme digestion that distinguished the cleavable PKM2 amplicon from the uncleavable PKM1 product. As shown in Fig 1C, in three representative normal brain samples, PKM1 transcripts clearly outnumbered PKM2 transcripts, with the ratio of PKM1 to PKM2 mRNA expression comparable to that determined by quantitative PCR in Fig 1A. Conversely, in grade IV astrocytomas, PKM2 transcripts outnumbered PKM1 transcripts by a 3:1 margin, comparable to that noted in quantitative PCR data. As a whole these results suggest that at the RNA level, high levels of PKM2 expression distinguish grade IV GBM from the other grades of glioma.

**Figure 2:**
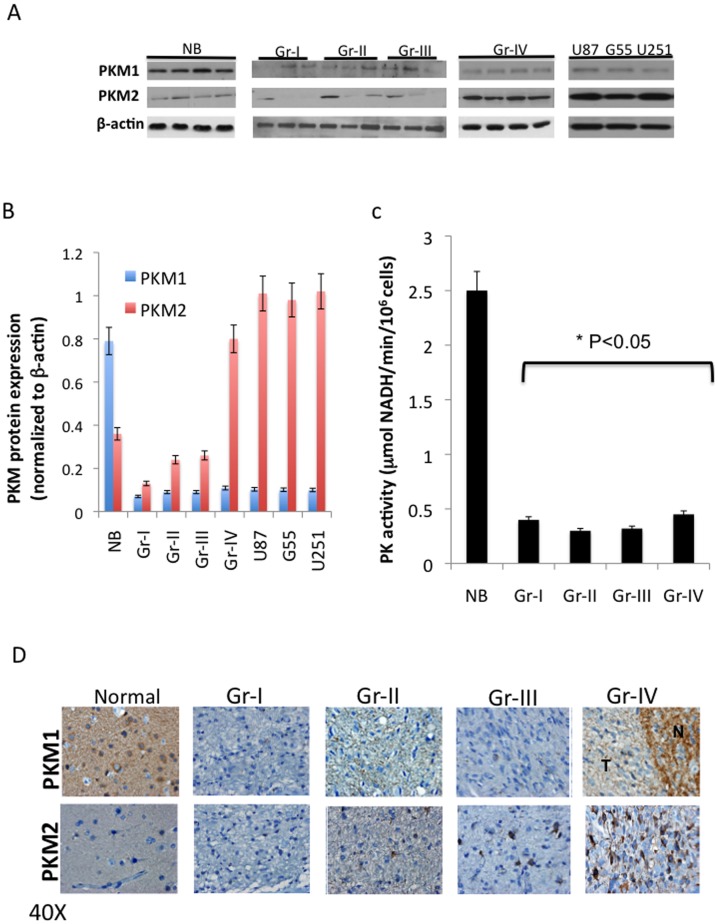
PKM1 and PKM2 protein expression in a series of WHO grade I-IV human astrocytoma specimens. **A**, Protein isolated from frozen normal brain (NB), grade (Gr) I-IV astrocytoma, and human GBM cell lines (U87, G55, U251) was subjected to Western blot analysis using PKM1-, PKM2-, and β-actin-specific antibodies. **B**, PKM signal intensity values derived from A and normalized to β-actin. **C**, IHC analysis of representative fixed sections from tumors in panel A using PKM1- or PKM2-specific antibodies. For Gr-IV sections T = tumor, N = tumor-infiltrated normal brain. **D**, Pyruvate kinase activity (mean±SE) determined using an LDH-coupled enzyme assay, N = 5 for each group.

To determine if the changes in PKM isoform expression noted at the RNA level were reflected in PKM protein expression and PK activity, fixed material and lysates from frozen samples used for RNA analysis were subjected to Western blot and immunohistochemical analysis using PKM1- and PKM2-selective antibodies, as well as to a biochemical assay of PK activity. As shown in the Western blots in Figs 2A and 2B, representative normal brain positive control samples expressed significantly more PKM1 protein than brain tumor samples or commonly used GBM cell lines, consistent with previously reported data [21]. These results were also consistent with immunohistochemical analyses of fixed tissue (Fig 2C), which showed that as noted at the RNA level, normal brain expresses higher levels of PKM1 protein than all gliomas. Consistent with the RNA analysis, levels of PKM1 protein expression were not significantly between the various classes of glioma. In contrast, and consistent with the RNA analyses presented, GBM and GBM cell lines expressed significantly more PKM2 protein than the other lower grade tumors or normal brain (Fig. 2A–C). These results therefore show that at both the RNA and protein levels, GBM appear different from lower grade glioma in their high level expression of PKM2.

Given the differences in PKM expression and aggressiveness of GBM relative to lower grade tumors, and the link between PKM isoform expression and metabolism, we also determined if changes in PK activity were noted across glioma grades. Consistent with the Western blot and immunohistochemical analyses, the PKM1-expresing normal brain samples exhibited high levels of PK activity while the PK activity of the astrocytomas, which expressed lower levels of the constitutively active PKM1, was uniformly lower than that noted in normal brain regardless of PKM2 expression (Fig. 2D). Of note, the grade IV astrocytomas had a PK activity that was not statistically different from that of glioma of other grades. These results therefore show that 1) normal brain expresses PKM1 mRNA and protein, but less PKM2 mRNA and protein, which is in turn linked to the high PK activity; 2) all astrocytomas ranging from benign pilocytic astrocytomas to grade IV GBM exhibit a significant decrease in PKM1 protein expression relative to normal brain, which in turn is linked to a low PK activity; 3) GBM are unique in that they exhibit an increase in PKM2 mRNA and protein expression relative to all other grades of glioma.

### PKM1 and PKM2 play key roles in the growth of glioma cells

Because all astrocytomas exhibit low level expression of PKM1 and low PK activity (relative to normal brain), and because GBM also up-regulate PKM2 expression, both of these events may be critical to glioma growth. To address this possibility, we modulated the expression of PKM1 and PKM2 and monitored the effect on PK activity, metabolic parameters, and the *in vitro* and *in vivo* growth of the cells. For these studies U87 and T98 GBM cells were used as these cells are both widely used and virtually indistinguishable in terms of PKM1/2 mRNA and protein expression from the primary and secondary GBM samples analyzed (Fig 1A and 2A).

**Figure 3:**
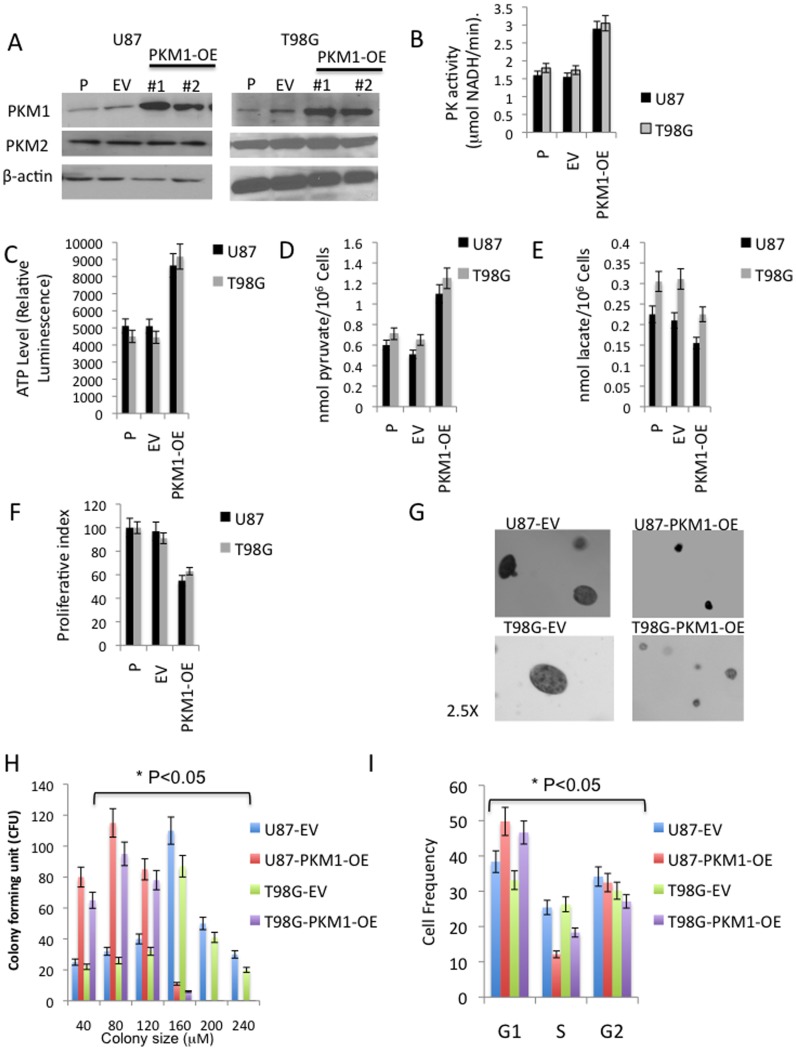
Over-expression of PKM1 alters metabolism and suppresses the growth of GBM cells. **A**, Parental U87 or T98 GBM cells were transfected with an empty vector (EV) or a construct encoding human PKM1. Two clonal populations (PKM1-1 and PKM1-2) were then established and analyzed by Western blot for levels of PKM1, PKM2, and β-actin. **B-F,** pyruvate kinase activity, ATP levels, intracellular concentrations of pyruvate and lactate, and proliferation index of cells from panel A. **G**, photomicrographs (2.5X magnification) of colonies generated by the 24th day growth of EV and PKM1-1 cells in soft agar. **H**, Colony number and distribution of colony size from experiments in (G). **I**, FACS-based cell cycle distribution of logarithmically-growing EV and PKM1-1 cells.

As shown in the Western blot in Fig 3A, introduction of a mammalian expression vector encoding PKM1 resulted in two independent populations of GBM cells in each cell line, each of which exhibited significant increases in PKM1 expression, but no change in PKM2 expression, relative to empty vector (EV) controls. Increased PKM1 expression was associated with an increase in PK activity relative to normal brain levels (Fig 3B, Fig 2D), as well as alterations in several metabolic parameters. Specifically, expression of PKM1 increased the intracellular levels of ATP (Fig 3C) and pyruvate (Fig 3D) and decreased the intracellular levels of lactate (Fig 3E), consistent with the ability of the constitutively active PKM1 isoform to convert PEP to pyruvate and to direct this pyruvate away from lactate production and toward ATP production in the mitochondria. Forced expression of PKM1 also had significant effects on the growth of the GBM cells; PKM1-expressing U87 and T98 cells proliferated more slowly in culture (Fig 3F), formed smaller colonies in soft agar (Fig 3 G, H), and exhibited an accumulation of cells with a G1 DNA content and a decrease in cells in S phase (Fig 3I). These results suggest that down-regulation of PKM1 expression is important for GBM cell growth, perhaps related to PKM1-mediated effects on cellular metabolism.

**Figure 4:**
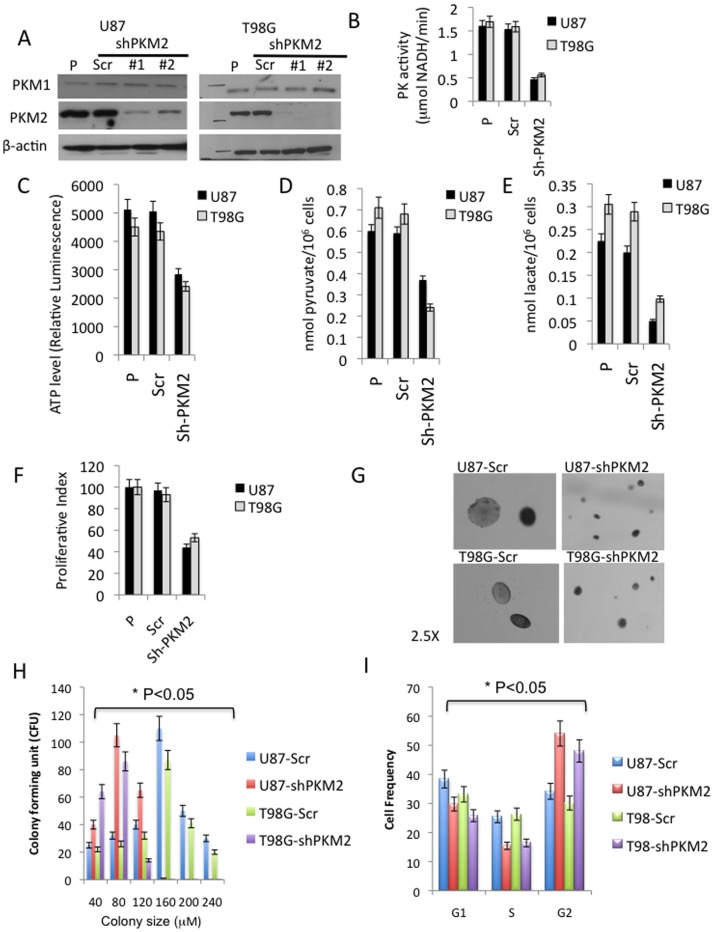
Suppression of PKM2 levels alters metabolism and reduces the growth of GBM cells. **A**, Parental U87 and T98 GBM cells were infected with lentivirus containing scramble shRNA (Scr) or one of five shRNAs targeting PKM2. Clonal populations (shPKM2-1 and shPKM2-2) were then established and analyzed by Western blot for levels of PKM1, PKM2, and β-actin. **B-F**, Pyruvate kinase activity, ATP levels, intracellular concentrations of pyruvate and lactate, and proliferation index of cells from panel A. **G**, photomicrographs (2.5X magnification) of colonies generated by the 24th day growth of Scr and shPKM2-1 cells in soft agar. **H**, Colony number and distribution of colony size from experiments in (G). **I**, FACS-based cell cycle distribution of logarithmically-growing Scr and PKM2-1 cells.

**Figure 5:**
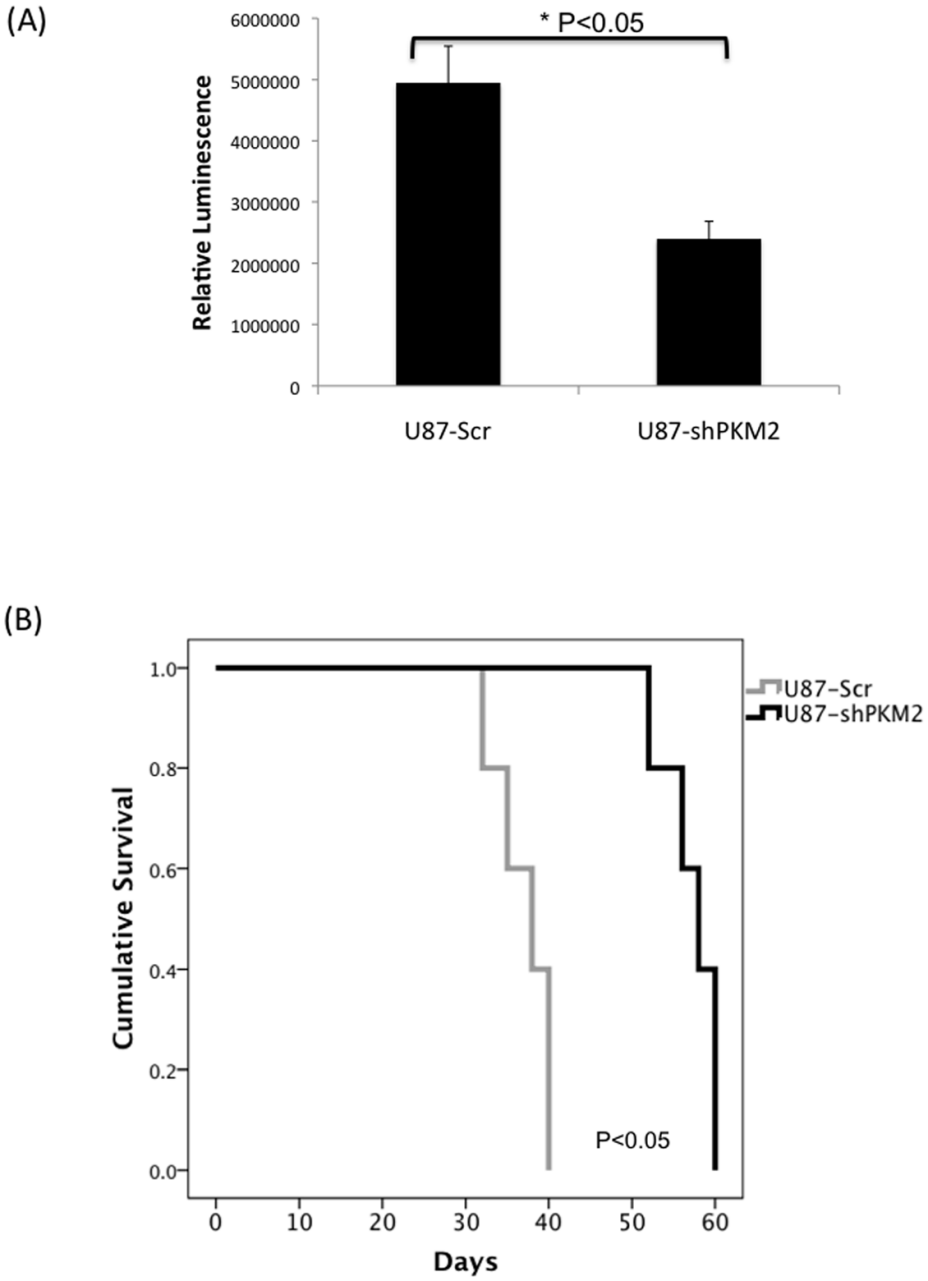
Suppression of PKM2 levels reduces the *in vivo* growth of GBM cells. **A**, cells derived in Fig 4A were luciferase modified, implanted intracranially into mice, and monitored for cell growth by bioluminescence imaging. Luminescence was normalized to day-1 post injection values, data presented is the mean of 5 animals at 24 days post implantation. **B**, Kaplan-Meyer survival curves for animals (N = 5 for each group) intracranially implanted with Scr or shPKM2-1 cells.

Because GBM up-regulate PKM2, and because suppression of PKM2 levels has been shown to inhibit tumor cell growth in other systems [6], [27] we also examined the consequences of PKM2 knock-down in GBM cells. As shown in the Western blot in Fig 4A, the lentiviral introduction of two different shRNAs targeting PKM2 resulted in two independent populations of GBM cells for each cell line, each of which exhibited significant decreases in PKM2 expression, but no change in PKM1 expression, relative to parental (P) and scramble (Scr) controls. Decreased PKM2 expression was associated with a significant decrease in PK activity (Fig. 4B), suggesting that most of the PK activity in these PKM1-deficient cells was the result of the small amount of PKM2 retained in the tetrameric, active form in these cells. Decreased PKM2 levels and the accompanying decrease in PK activity was associated with decreased intracellular levels of ATP, pyruvate, and lactate (Fig 4C–E), consistent with the loss of PK activity leading to decreased conversion of PEP to pyruvate and decreased pyruvate available for conversion to lactate or for ATP production. The decrease in PK activity and ATP generation resulting from suppression of PKM2 levels would be expected to favor tumor cell growth based on the macromolecular synthesis argument previously noted. PKM2, however, also has activities that allow it, even in the metabolically inactive dimeric form, to stimulate growth [28], [29], and consistent with these observations, PKM2 knock-down cells proliferated more slowly in culture (Fig 4F), formed fewer and smaller colonies in soft agar (Fig 4 G-H), formed tumors that were smaller than those formed by control cells 24 days following intracranial implantation (Fig 5A), and took significantly longer to result in symptoms that necessitated sacrifice of the animals (Fig 5B). In contrast to previously published data, however [28], both GBM cell lines in which PKM2 levels were suppressed exhibited an accumulation of G2/M cells based on their 4n DNA content (Fig 4I) as well as an increase in expression of enzymes associated with G2 arrest (cdc25c, cyclin B, not shown) relative to scramble controls. These results show that PKM2 over-expression in GBM contributes to continued tumor growth in ways consistent with the known non-metabolic activities of the protein, and that both the up-regulation of PKM2 expression and down-regulation of PKM1 expression and PK activity noted in all grades of glioma play important roles in glioma cell growth.

## Discussion

In our examination of PKM isoform expression and PK activity in a series of over 100 astrocytomas, we found that PKM1 expression and PK activity was consistently low (relative to normal brain tissue) across as wide-range of gliomas, but that PKM2 expression increased in a GBM-specific manner. Both low levels of PKM1/PK activity and high levels of PKM2 were, however, critical for continued growth of glioma cells.

Numerous groups have demonstrated that GBM express more PKM2 than normal brain, although the extent to which PKM expression and PK activity vary with respect to glioma grade has only partially and indirectly been examined. David et al examined the ratio of PKM2 to PKM1 RNA expression in 4 grade I gliomas, 4 low-grade astrocytomas, and 4 GBM [21]. The results of these studies agree with the present data in showing that all glioma express more PKM2 mRNA than PKM1 mRNA. The methodology in the David study, however, did not allow a direct comparison of PKM2 expression or PK activity between the tumor groups, and as such could not evaluate how these changes with regard to glioma grade. Similarly, Chinnaiyan et al compared PK activity and PKM2 protein expression in groups of grade 3 and 4 glioma sub-typed by expression profiling into proneural or mesenchymal sub-groups [30]. Although the proneural glioma were suggested to have higher levels of PK activity and lower levels of PKM2 protein expression than the mesenchymal tumors, grade 3 and grade 4 tumors were represented in both groups, precluding both comparison to the present data and any conclusions about PKM expression and activity based on tumor grade. The present studies therefore represent the first complete analysis of PKM expression and PK activity with regard to glioma grade, and are the first to our knowledge to show grade-specific differences in PKM2 expression, but not PK activity, in glioma.

One of the most striking observations of the present study is the significant up-regulation of PKM2 expression in GBM relative to all other forms of glioma. Previous studies have suggested that even slow-growing benign oncocytoma exhibit high levels of PKM2 expression, and pre-malignant Barrett's intestinal metaplasia also exhibited significant PKM2 over-expression [11], [31]. It was therefore surprising that benign grade I glioma exhibited levels of PKM2 expression no different from those of frankly malignant grade III glioma, but less than that of GBM. A trivial explanation of this data is that the grade I-III tumor samples, as a result of the diffuse nature of the tumors, contain a larger percentage of normal cells than the grade IV tumors, and that the admixture of normal cells expressing high levels of PKM1 and tumor cells expressing high levels of PKM2 results in a grade I-III glioma sample that appears as a whole to have a lower level of PKM2 expression than its truly tumorigenic components. All samples used, however, were verified by pathologic examination to contain >90% tumor, and even if this explanation were true, mixed samples would be expected to have higher levels of both PKM1 and PKM2, rather than just the increased levels of PKM2 (relative to normal brain) noted. The related question of whether all glioma over-express PKM2 is an open to interpretation and based on the point of reference. Relative to normal brain, all glioma express higher levels of PKM2 and lower levels of PKM1. These tumors, however, most likely did not arise from normal differentiated brain cells and more likely arose from a stem or progenitor population [32], [33]. Further complicating the matter, each grade of glioma may arise from a distinct progenitor population, and even with a given grade there may be different cells of origin [34]. Nonetheless, the pattern of PKM1/PKM2 expression in grade I-III glioma more closely resembles that of NSC population than normal brain. Whether these tumors represent a frozen stage of differentiation or a dramatic shift in metabolic profile remains an unanswerable question. It is clear from the data, however, that GBM represent a unique type of glioma, quantitatively different from the other grades of glioma with respect to PKM2 expression.

If GBM exhibit significantly higher levels of PKM2 expression than other glioma, one important question is how this grade-specific up-regulation occurs. A number of genetic events including c-myc over-expression, growth factor over-expression, and Ras pathway activation have been linked to the control of PKM transcript splicing [21], [35]-[38], and the increased incidence of these alterations in GBM may tilt the balance of splicing toward production of the PKM2 transcript and help explain the grade-specific increases in PKM2 expression noted. It's worth noting, however, that the present study examined both *de novo* GBM, as well as secondary GBM that arose from lower grade tumors based on their mutant IDH status. The fact that both groups of GBM exhibit increased PKM2 expression despite their very different genetic compositions [39] suggests that the up-regulation of PKM2 in GBM appears to be driven by fundamental processes shared by all GBM rather than by known genetic alterations that play a direct role in the PKM splicing process but differ between the two GBM groups analyzed.

The work presented also suggests that modulation of both PKM expression and PK activity are important for continued glioma cell growth. The GBM cells used for these studies were representative of primary GBM in terms of patterns of PKM isoform expression and PK activity, and in these cells the up-regulation of PKM1 in the face of high endogenous levels of PKM2 increased PKM activity and suppressed growth, suggesting that optimal glioma growth is limited by high PK activity. Although the basis for this effect is unclear, the data are consistent with idea that the use of glucose for pyruvate and ATP production limits the ability of cells to create the macromolecules needed for increased proliferation [40], resulting in a generalized accumulation of cells G0/G1. The modulation of the end products of PK activity may therefore be a reasonable strategy to inhibit growth of these GBM cells. In contrast, suppression of PKM2 levels in the face of low PKM1 levels also suppressed cell growth. Similar findings in other systems were attributed to levels of PK activity insufficient to sustain growth [6], [40], although in the present study the PK activity levels in the PKM2 knock-down cell were comparable to those in primary GBM. An alternative explanation may be provided by recent studies showing that PKM2 not only has metabolic effects but can also translocate to the nucleus and facilitate cyclin D1 expression and cell cycle progression [28]. While the effects of PKM2 knock-down noted in the present study are consistent with the non-metabolic effects of PKM2, the accumulation of cells was not associated with a generalized slowing in cell cycle progression but rather a specific G2 arrest. It may therefore be possible that PKM2 has non-metabolic effects on regulators of the G2 checkpoint that cooperate with, or supersede, those related to cyclin D1.

The present findings provide the first detailed picture of PKM expression and activity across a range of gliomas of different grades. These studies provide a more complex picture of PKM isoform regulation than previously described, and suggest that while PK activity is uniform across all grades of glioma, PKM2 expression is up-regulated not at the benign/malignant transition and not in a gradual manner related to tumor grade, but rather most dramatically in GBM. Therapeutic approaches targeting metabolic changes in glioma may therefore benefit from considering glioma subtypes separately, and in particular in focusing on the potentially unique role of PKM2 over-expression in GBM.
